# Correction: Debel et al. Does the Choice of Anaesthesia Affect Cancer? A Molecular Crosstalk between Theory and Practice. *Cancers* 2023, *15*, 209

**DOI:** 10.3390/cancers17182941

**Published:** 2025-09-09

**Authors:** Wiebrecht Debel, Ali Ramadhan, Caroline Vanpeteghem, Ramses G. Forsyth

**Affiliations:** 1Department of Anesthesiology, University Hospital Ghent, 9000 Ghent, Belgium; wiebrecht.debel@ugent.be (W.D.); caroline.vanpeteghem@ugent.be (C.V.); 2Department of Pathological Anatomy, Universitair Ziekenhuis Brussel, 1090 Brussels, Belgium; ali.ramadhan@uzbrussel.be; 3Laboratorium for Experimental Pathology (EXPA), Vrije Universiteit Brussel, 1090 Brussels, Belgium

## References

In the original publication [1], The following publications were removed due to questionable data together with the presence of a Pubpeer notification (see also: https://pubpeer.org/publications/1E84D6C02544F2EB4C8F06E244B39B): Xia, M.; Ji, N.N.; Duan, M.L.; Tong, J.-H.; Xu, J.-G.; Zhang, Y.-M.; Wang, S.-H. Dexmedetomidine regulate the malignancy of breast cancer cells by activating α2-adrenoceptor/ERK signaling pathway. *Eur. Rev. Med. Pharmacol. Sci.*
**2016**, *20*, 3500–3506. (original reference 10)Huang, H.; Benzonana, L.L.; Zhao, H.; Watts, H.R.; Perry, N.J.S.; Bevan, C.L.; Brown, R.; Ma, D. Prostate cancer cell malignancy via modulation of HIF-1α pathway with isoflurane and propofol alone and in combination. *Br. J. Cancer* **2014**, *111*, 1338–1349, https://doi.org/10.1038/bjc.2014.426. (original reference 19)Yang, X.; Zheng, Y.-T.; Rong, W. Sevoflurane induces apoptosis and inhibits the growth and motility of colon cancer in vitro and in vivo via inactivating Ras/Raf/MEK/ERK signaling. *Life Sci.* **2019**, *239*, 116916, https://doi.org/10.1016/j.lfs.2019.116916. (original reference 22)He, J.; Zhao, H.; Liu, X.; Wang, D.; Wang, Y.; Ai, Y.; Yang, J. Sevoflurane suppresses cell viability and invasion and promotes cell apoptosis in colon cancer by modulating exosome-mediated circ-HMGCS1 via the miR-34a-5p/SGPP1 axis. *Oncol. Rep.* **2020**, *44*, 2429–2442, https://doi.org/10.3892/or.2020.7783. original reference 23)Sun, S.Q.; Ren, L.J.; Liu, J.; Wang, P.; Shan, S.M. Sevoflurane inhibits migration and invasion of colorectal cancer cells by regulating microRNA-34a/ADAM10 axis. *Neoplasma* **2019**, *66*, 887–895, https://doi.org/10.4149/neo_2018_181213n962. (original reference 24)Fan, L.; Wu, Y.; Wang, J.; He, J.; Han, X. Sevoflurane inhibits the migration and invasion of colorectal cancer cells through regulating ERK/MMP-9 pathway by up-regulating miR-203. *Eur. J. Pharmacol.* **2019**, *850*, 43–52, https://doi.org/10.1016/j.ejphar.2019.01.025. (original reference 25)Zhang, M.W.; Sheng, M.B.; Chen, M.S.; Zhao, B.H.; Wu, B.L.; Sun, M.Y.; Cui, B.J.; Zhu, M.X.; Ma, M.D. Sevoflurane Enhances Proliferation, Metastatic Potential of Cervical Cancer Cells *via* the Histone Deacetylase 6 Modulation In Vitro. *Anesthesiology* **2020**, *132*, 1469–1481, https://doi.org/10.1097/aln.0000000000003129. (original reference 29)Xu, W.; Xue, R.; Xia, R.; Liu, W.-W.; Zheng, J.-W.; Tang, L.; Kang, L.-Y.; Wang, W.; Wei W.-T. Sevoflurane impedes the progression of glioma through modulating the circular RNA has_circ_0012129/miR-761/TGIF2 axis. *Eur. Rev. Med. Pharmacol. Sci.*
**2020**, *24*, 5534–5548. https://doi.org/10.26355/eurrev_202005_21339. (original reference 34)Meng, C.; Song, L.; Wang, J.; Li, D.; Liu, Y.; Cui, X. Propofol induces proliferation partially via downregulation of p53 protein and promotes migration via activation of the Nrf2 pathway in human breast cancer cell line MDA-MB-231. *Oncol. Rep.* **2016**, *37*, 841–848, https://doi.org/10.3892/or.2016.5332. (original reference 42)Yang, N.; Liang, Y.; Yang, P.; Ji, F. Propofol suppresses LPS-induced nuclear accumulation of HIF-1α and tumor aggressiveness in non-small cell lung cancer. *Oncol. Rep.* **2017**, *37*, 2611–2619, https://doi.org/10.3892/or.2017.5514. (original reference 43)Du, Q.; Liu, J.; Zhang, X.; Zhang, X.; Zhu, H.; Wei, M.; Wang, S. Propofol inhibits proliferation, migration, and invasion but promotes apoptosis by regulation of Sox4 in endometrial cancer cells. *Braz. J. Med Biol. Res.* **2018**, *51*, e6803, https://doi.org/10.1590/1414-431x20176803. (original reference 44)Zhou, C.-L.; Li, J.-J.; Ji, P. Propofol Suppresses Esophageal Squamous Cell Carcinoma Cell Migration and Invasion by Down-Regulation of Sex-Determining Region Y-box 4 (SOX4). *J. Pharmacol. Exp. Ther.* **2017**, *23*, 419–427, https://doi.org/10.12659/msm.899732. (original reference 45)Ye, Z.; Jingzhong, L.; Yangbo, L.; Lei, C.; Jiandong, Y. Propofol Inhibits Proliferation and Invasion of Osteosarcoma Cells by Regulation of MicroRNA-143 Expression. *Oncol. Res. Featur. Preclin. Clin. Cancer Ther.* **2014**, *21*, 201–207, https://doi.org/10.3727/096504014x13890370410203. (original reference 46)Wu, K.-C.; Yang, S.-T.; Hsia, T.-C.; Yang, J.-S.; Chiou, S.-M.; Lu, C.-C.; Wu, R.S.-C.; Chung, J.-G. Suppression of cell invasion and migration by propofol are involved in down-regulating matrix metalloproteinase-2 and p38 MAPK signaling in A549 human lung adenocarcinoma epithelial cells. *Anticancer Res.*
**2012**, *32*, 4833–4842. (original reference 47)Huang, X.; Teng, Y.; Yang, H.; Ma, J. Propofol inhibits invasion and growth of ovarian cancer cells via regulating miR-9/NF-κB signal. *Braz. J. Med Biol. Res.* **2016**, *49*, e5717, https://doi.org/10.1590/1414-431x20165717. (original reference 48)Miao, Y.; Zhang, Y.; Wan, H.; Chen, L.; Wang, F. GABA-receptor agonist, propofol inhibits invasion of colon carcinoma cells. *Biomed. Pharmacother.* **2010**, *64*, 583–588, https://doi.org/10.1016/j.biopha.2010.03.006. (original reference 50)Peng, Z.; Zhang, Y. Propofol inhibits proliferation and accelerates apoptosis of human gastric cancer cells by regulation of microRNA-451 and MMP-2 expression. *Genet. Mol. Res.* **2016**, *15*, https://doi.org/10.4238/gmr.15027078. (original reference 51)Xu, J.; Xu, W.; Zhu, J. Propofol suppresses proliferation and invasion of glioma cells by upregulating microRNA-218 expression. *Mol. Med. Rep.* **2015**, *12*, 4815–4820, https://doi.org/10.3892/mmr.2015.4014. (original reference 52)Liu, F.; Qiu, F.; Fu, M.; Chen, H.; Wang, H. Propofol Reduces Epithelial to Mesenchymal Transition, Invasion and Migration of Gastric Cancer Cells through the MicroRNA-195-5p/Snail Axis. *J. Pharmacol. Exp. Ther.* **2020**, *26*, e920981–e920981-12, https://doi.org/10.12659/msm.920981. (original reference 53)Zhang, Y.; Li, C.; Zhou, Y.; Lu, X. Effects of propofol on colon cancer metastasis through STAT3/HOTAIR axis by activating WIF-1 and suppressing Wnt pathway. *Cancer Med.* **2020**, *9*, 1842–1854, https://doi.org/10.1002/cam4.2840. (original reference 54)Cui, W.-Y.; Liu, Y.; Zhu, Y.-Q.; Song, T.; Wang, Q.-S. Propofol induces endoplasmic reticulum (ER) stress and apoptosis in lung cancer cell H460. *Tumor Biol.* **2014**, *35*, 5213–5217, https://doi.org/10.1007/s13277-014-1677-7. (original reference 55)Gong, T.; Ning, X.; Deng, Z.; Liu, M.; Zhou, B.; Chen, X.; Huang, S.; Xu, Y.; Chen, Z.; Luo, R. Propofol-induced miR-219-5p inhibits growth and invasion of hepatocellular carcinoma through suppression of GPC3-mediated Wnt/β-catenin signalling activation. *J. Cell. Biochem.* **2019**, *120*, 16934–16945, https://doi.org/10.1002/jcb.28952. (original reference 56)Du, Y.; Zhang, X.; Zhang, H.; Chen, Y.; Zhu, S.; Shu, J.; Pan, H. Propofol modulates the proliferation, invasion and migration of bladder cancer cells through the miR-145-5p/TOP2A axis. *Mol. Med. Rep.* **2021**, *23*, 1–11, https://doi.org/10.3892/mmr.2021.12078. (original reference 57)Liu, Y.-P.; Heng, J.-Y.; Zhao, X.-Y.; Li, E.-Y. The inhibition of circular RNA circNOLC1 by propofol/STAT3 attenuates breast cancer stem cells function via miR-365a-3p/STAT3 signaling. *J. Transl. Med.* **2021**, *19*, 1–16, https://doi.org/10.1186/s12967-021-03133-5. (original reference 59)Yu, B.; Gao, W.; Zhou, H.; Miao, X.; Chang, Y.; Wang, L.; Xu, M.; Ni, G. Propofol induces apoptosis of breast cancer cells by downregulation of miR-24 signal pathway. *Cancer Biomarkers* **2018**, *21*, 513–519, https://doi.org/10.3233/CBM-170234. (original reference 62)Liu, Z.; Zhang, J.; Hong, G.; Quan, J.; Zhang, L.; Yu, M. Propofol inhibits growth and invasion of pancreatic cancer cells through regulation of the miR-21/Slug signaling pathway. *Am. J. Transl. Res*. **2016**, *8*, 4120–4133. (original reference 63)Yang, N.; Liang, Y.; Yang, P.; Yang, T.; Jiang, L. Propofol inhibits lung cancer cell viability and induces cell apoptosis by upregulating microRNA-486 expression. *Braz. J. Med Biol. Res.* **2017**, *50*, e5794, https://doi.org/10.1590/1414-431x20165794. (original reference 64)Zheng, X.; Dong, L.; Zhao, S.; Li, Q.; Liu, D.; Zhu, X.; Ge, X.; Li, R.; Wang, G. Propofol Affects Non–Small-Cell Lung Cancer Cell Biology By Regulating the miR-21/PTEN/AKT Pathway In Vitro and In Vivo. *Obstet. Anesthesia Dig.* **2020**, *131*, 1270–1280, https://doi.org/10.1213/ane.0000000000004778. (original reference 66)Wang, H.; Jiao, H.; Jiang, Z.; Chen, R. Propofol inhibits migration and induces apoptosis of pancreatic cancer PANC-1 cells through miR-34a-mediated E-cadherin and LOC285194 signals. *Bioengineered* **2020**, *11*, 510–521, https://doi.org/10.1080/21655979.2020.1754038. (original reference 67)Sun, H.; Gao, D. Propofol suppresses growth, migration and invasion of A549 cells by down-regulation of miR-372. *BMC Cancer* **2018**, *18*, 1–11, https://doi.org/10.1186/s12885-018-5175-y. (original reference 68)Zhao, H.; Wei, H.; He, J.; Wang, D.; Li, W.; Wang, Y.; Ai, Y.; Yang, J. Propofol disrupts cell carcinogenesis and aerobic glycolysis by regulating circTADA2A/miR-455-3p/FOXM1 axis in lung cancer. *Cell Cycle* **2020**, *19*, 2538–2552, https://doi.org/10.1080/15384101.2020.1810393. (original reference 73)Song, F.; Liu, J.; Feng, Y.; Jin, Y. Propofol-induced HOXA11-AS promotes proliferation, migration and invasion, but inhibits apoptosis in hepatocellular carcinoma cells by targeting miR-4458. *Int. J. Mol. Med.* **2020**, *46*, 1135–1145, https://doi.org/10.3892/ijmm.2020.4667. (original reference 75)Chen, X.; Wu, Q.; You, L.; Chen, S.; Zhu, M.; Miao, C. Propofol attenuates pancreatic cancer malignant potential via inhibition of NMDA receptor. *Eur. J. Pharmacol.* **2017**, *795*, 150–159, https://doi.org/10.1016/j.ejphar.2016.12.017. (original reference 76)Wang, Z.; Gong, H.; Zheng, F.; Liu, D.; Dong, T. Propofol suppresses proliferation and invasion of pancreatic cancer cells by upregulating microRNA-133a expression. *Genet. Mol. Res.* **2015**, *14*, 7529–7537, https://doi.org/10.4238/2015.july.3.28. (original reference 82)Buckley, A.; McQuaid, S.; Johnson, P.; Buggy, D.J. Effect of anaesthetic technique on the natural killer cell anti-tumour activity of serum from women undergoing breast cancer surgery: a pilot study. *Br. J. Anaesth.* **2014**, *113*, i56–i62, https://doi.org/10.1093/bja/aeu200. (original reference 98)Friesen, C.; Roscher, M.; Hormann, I.; Fichtner, I.; Alt, A.; Hilger, R.A.; Debatin, K.-M.; Miltner, E. Cell death sensitization of leukemia cells by opioid receptor activation. *Oncotarget* **2013**, *4*, 677–690, https://doi.org/10.18632/oncotarget.952. (original reference 103)Wang, H.-W.; Wang, L.-Y.; Jiang, L.; Tian, S.-M.; Zhong, T.-D.; Fang, X.-M. Amide-linked local anesthetics induce apoptosis in human non-small cell lung cancer. *J. Thorac. Dis.* **2016**, *8*, 2748–2757, https://doi.org/10.21037/jtd.2016.09.66. (original reference 108)Chen, J.; Jiao, Z.; Wang, A.; Zhong, W. Lidocaine inhibits melanoma cell proliferation by regulating ERK phosphorylation. *J. Cell. Biochem.* **2018**, *120*, 6402–6408, https://doi.org/10.1002/jcb.27927. (original reference 111)Xuan, W.; Zhao, H.; Hankin, J.; Chen, L.; Yao, S.; Ma, D. Local anesthetic bupivacaine induced ovarian and prostate cancer apoptotic cell death and underlying mechanisms in vitro. *Sci. Rep.* **2016**, *6*, 26277, https://doi.org/10.1038/srep26277. (original reference 112)Chang, Y.-C.; Hsu, Y.-C.; Liu, C.-L.; Huang, S.-Y.; Hu, M.-C.; Cheng, S.-P. Local Anesthetics Induce Apoptosis in Human Thyroid Cancer Cells through the Mitogen-Activated Protein Kinase Pathway. *PLOS ONE* **2014**, *9*, e89563, https://doi.org/10.1371/journal.pone.0089563. (original reference 113)Jiang, Y.; Gou, H.; Zhu, J.; Tian, S.; Yu, L. Lidocaine inhibits the invasion and migration of TRPV6-expressing cancer cells by TRPV6 downregulation. *Oncol. Lett.* **2016**, *12*, 1164–1170, https://doi.org/10.3892/ol.2016.4709. (original reference 119)Sun, H.; Sun, Y. Lidocaine inhibits proliferation and metastasis of lung cancer cell via regulation of miR-539/EGFR axis. *Artif. Cells, Nanomedicine, Biotechnol.* **2019**, *47*, 2866–2874, https://doi.org/10.1080/21691401.2019.1636807. (original reference 121)Gao, H.; Chakraborty, G.; Lee-Lim, A.P.; Mo, Q.; Decker, M.; Vonica, A.; Shen, R.; Brogi, E.; Brivanlou, A.H.; Giancotti, F.G. The BMP Inhibitor Coco Reactivates Breast Cancer Cells at Lung Metastatic Sites. *Cell* **2012**, *150*, 764–779, https://doi.org/10.1016/j.cell.2012.06.035. (original reference 134)

In addition to the removal of several references, minor corresponding adjustments were made in the text and tables to ensure consistency.

The authors apologize for any inconvenience caused and state that the scientific conclusions are unaffected. They emphasize the need for more transparent study protocols in the available literature, including precise reporting of drug dosage and duration, to enable better study design and more accurate evaluation of the impact on cancer outcomes during surgery. This correction was approved by the Academic Editor. The original publication has also been updated.
